# Determination of Antibacterial and Antioxidant Potential of Some Medicinal Plants from Saurashtra Region, India

**DOI:** 10.4103/0250-474X.57289

**Published:** 2009

**Authors:** M. Kaneria, Y. Baravalia, Y. Vaghasiya, S. Chanda

**Affiliations:** Phytochemical, Pharmacological and Microbiological Laboratory, Department of Biosciences, Saurashtra University, Rajkot-360 005, India

**Keywords:** Antibacterial activity, antioxidant activity, *Guazuma ulmifolia* L., *Manilkara zapota* L., *Melia azedarach* L.

## Abstract

Many plants used in Saurashtra folk medicine have been reported to exhibit high antibacterial and antioxidant activities. In the present study, some parts of five plants, *Guazuma ulmifolia* L., *Manilkara zapota* L., *Melia azedarach* L., *Syzygium cumini* L. and *Wrightia tomentosa* R.& S., were evaluated for their antibacterial activity, total phenol content, flavonoid content, 2,2-diphenyl-1-picrylhydrazyl free radical scavenging activity and phytochemical analysis, using successive extraction by cold percolation method with petroleum ether, ethyl acetate, methanol and water. *In vitro* antibacterial activity was evaluated against five bacterial strains viz. *Bacillus subtilis, Staphylococcus aureus, Pseudomonas aeruginosa, Salmonella typhimurium* and *Enterobacter aerogenes* by agar well diffusion method. Among the plants screened, *W. tomentosa* leaf and fruit showed the best antibacterial activity. The Gram-positive bacteria were more susceptible than Gram-negative bacteria. Methanol extract of *M. zapota* showed the best 2,2-diphenyl-1-picrylhydrazyl free radical scavenging activity. Highest total phenol content was shown by *M. zapota* and *S. cumini* in methanol extract, while highest flavonoid content was shown by *W. tomentosa* stem in petroleum ether extract and ethyl acetate extract. In all the plants, cardiac glycosides and triterpenes were more as compared to other phytoconstituents.

The frequency of life-threatening infections caused by pathogenic microorganisms has increased worldwide and is becoming an important cause of morbidity and mortality in immuno compromised patients in developed countries[1]. There is an urgent need to control antimicrobial resistance by improved antibiotic usage and reduction of hospital cross infection[2]; however the development of new antibiotics should be continued as they are of primary importance to maintain the effectiveness of antimicrobial treatment[3]. In developing countries, the World Health Organization[4] estimates that about three quarters of the population relies on plant based preparations used in their traditional medicinal system and as the basic needs for human primary health care. Therefore, several medicinal plants have been evaluated for possible antimicrobial activity and to get remedy for a variety of ailments of microbial origin[5,6].

Oxidative stress is an important contributor to the pathophysiology of a variety of pathological conditions including cardiovascular dysfunction, atherosclerosis, inflammation, carcinogenesis, drug toxicity, reperfusion injury and neurodegenerative diseases[7]. Plants (fruits, vegetables, medicinal herbs) contain a wide variety of free radical scavenging molecules, such as phenolic compounds, nitrogen compounds, vitamins, terpenoids and some other endogenous metabolites, that are rich in antioxidant activity[8–11]. Scavenging of free radicals by antioxidants could reduce the fibrosis process in the tissue[12]. Antioxidants are considered as possible protective agents reducing oxidative damage to the human body[13]. Antioxidants are naturally abundant in fruits and are able to neutralize free radicals donating an electron and converting them to harmless molecules[14].

Natural antioxidants have a wide range of biochemical activities, including inhibition of ROS generation, direct or indirect scavenging of free radicals, and alteration of intra cellular redox potential[15]. Antioxidants provide protection to living organisms from damage caused by uncontrolled production of reactive oxygen species and the concomitant lipid peroxidation, protein damage and DNA strand breakage[16]. An antioxidant, which can quench reactive free radicals, can prevent the oxidation of other molecules and may, therefore, have health promoting effects in the prevention of degenerative diseases[17]. In addition, it has been reported that there is an inverse relationship between dietary intake of antioxidant rich food and the incidence of human diseases[18].

Hence, in this article, antibacterial and antioxidant activities of the extracts obtained from five plants from their different parts (leaf, stem and fruit) that have been traditionally used as general health supplements are examined. The plants studied were *Guazuma ulmifolia* L. (Sterculiaceae), *Manilkara zapota* L. (Sapotaceae), *Melia azedarach* L. (Meliaceae),*Syzygium cumini* L. (Myrtaceae) and *Wrightia tomentosa* R.& S. (Apocynaceae). The aim of this current work was to evaluate the antibacterial and antioxidant potential of extracts in different solvents of all these plants, as well as their total phenol and flavonoid contents.

## MATERIALS AND METHODS

### Collection of plant material:

The plants were collected from Rajkot, Gujarat, India in September 2007 and identified at the Department of Biosciences, Saurashtra University, Rajkot, Gujarat, India. The ethnobotanical information of the screened plants is given in Table 1[19]. The plant parts were thoroughly washed with tap water, air dried, homogenized to fine powder and stored in air tight bottles.

**TABLE 1:** THE ETHNOBOTANICAL INFORMATION OF THE SCREENED PLANTS

Plant Species	Vernacular Name	Family	Parts used	Therapeutic uses
*Guazuma ulmifolia* L.	*Rudrakshi*	Sterculiaceae	Leaf	Bark tonic used as demulcent and elephantiasis.
*Manilkara zapota* L.	*Chiku*	Sapotaceae	Leaf	Bark is an antibiotic, astringent and febrifuge. Chicle from bark is used in dental surgery.
*Melia azedarach* L.	*Bakan Limdo*	Meliaceae	Leaf, Stem	The leaves are useful in hysteria, leprosy, cough, bronchitis and scabies.
*Syzygium cumini* L.	*Jambu*	Myrtaceae	Leaf	Bark, leaf, fruit used in diabetes, leucorrhoea, stomachalgia and gastropathy.
*Wrightia tomentosa R. & S.*	*Dudhlo*	Apocynaceae	Leaf, Stem, Fruit	Root, bark, seed, used in piles, fever colic, diarrhea and round worm

### Extraction:

The dried powder of plants was extracted successively in petroleum ether, ethyl acetate, methanol and distilled water by cold percolation method[20]. Ten grams of dried powder was taken in 100 ml of solvent in a conical flask, plugged with cotton wool and then kept on a rotary shaker at 190–220 rpm for 24 h. After 24 h, the extract was filtered with eight layers of muslin cloth; the residue was dried and used for extraction in another solvent while the filtrate was centrifuged at 5000 g for 10 min, the supernatant was collected and the solvent was evaporated. The dried extract of each solvent, was stored at 4° in airtight bottles. The extraction was done at least three times and the mean values are presented.

### Test microorganisms:

The bacterial strains used are identified strains and were obtained from National Chemical Laboratory, Pune, India. Two Gram-positive bacteria, *Bacillus subtilis* ATCC6633 and *Staphylococcus aureus* ATCC29737, and three Gram-negative bacteria, *Pseudomonas aeruginosa* ATCC27853, *Salmonella typhimurium* ATCC23564 and *Enterobacter aerogenes* ATCC13048 were studied.

### Antibacterial Assay:

A loop full of the strain was inoculated in 25 ml of nutrient broth in a conical flask and incubated at room temperature on a rotary shaker for 24 h to activate the test bacteria. The inoculum size was 1×10^8^ cells. Muller Hinton agar No. 2 (Himedia) was used for the antibacterial susceptibility study. The bacterial assay was performed by agar well diffusion method[21,22]. The media and the test bacterial cultures were poured into Petri dishes (Hi-Media). The test strain (200 μl) was inoculated into the media when the temperature reached 40-42°. Care was taken to ensure proper homogenization. After the media was solidified; a well was made in the plates with the help of a cup-borer (8.5 mm). The well was filled with 100 μl of the extract (500 μg/well) and the plates were incubated over night at 37°. The bacterial growth was determined by measuring the diameter of the zone of inhibition. The experiment was done three times and the mean values are presented. For each bacterial strain, controls were maintained where pure solvent (DMSO) was used instead of the extract.

### Phytochemical analysis:

The preliminary phytochemical screening of different extracts was done to ascertain the presence of bioactive components. The presence of alkaloids (dragendroff, Mayer, Wagner), flavonoids, tannins, phlobatannins, triterpenes, steroids, saponins and cardiac glycosides was determined[23,24].

### Total phenol determination:

Total phenolic content of the extracts was determined by Folin Ciocalteu reagent method[25] with some modifications. Plant extract (1 ml) was mixed with Folin Ciocalteu reagent (0.1 ml, 1 N), and allowed to stand for 15 min. Then 5 ml of saturated Na_2_CO_3_ was added. The mixtures were allowed to stand for 30 min at room temperature and the total phenols were determined spectrophotometrically at 760 nm. The calibration curve was prepared by preparing gallic acid (10–100 μg/ml) solution in distilled water. Total phenol values are expressed in terms of gallic acid equivalent (mg/g of extracted compound).

### Flavonoid determination:

Aluminum chloride colorimetric method[26] with some modifications was used to determine flavonoid content. Plant extract (1 ml) in methanol was mixed with 1 ml of methanol, 0.5 ml aluminum chloride (1.2%) and 0.5 ml potassium acetate (120 mM). The mixture was allowed to stand for 30 min at room temperature; the absorbance of the reaction mixture was measured at 415 nm. The calibration curve was prepared by preparing quercetin (5-60 μg/ml) solution in methanol. Flavonoid content is expressed in terms of quercetin equivalent (mg/g of extracted compound).

### 2,2-Diphenyl-1-picrylhydrazyl (DPPH) free radicals scavenging activity:

The free radical scavenging activity of the extracts of various plant species was measured using the modified method of McCune and Johns[27]. The extracts were dissolved in methanol. Various concentrations of the extracts (100-1000 μg) were added to DPPH solution in methanol (0.1 mM). The mixture was shaken vigorously and allowed to stand for 10 min at room temperature. The absorbance of the resulting solution was measured at 517 nm. The radical scavenger activity is expressed in terms of the amount of antioxidants necessary to decrease the initial DPPH absorbance by 50% (IC_50_).

## RESULTS AND DISCUSSION

The extractive yield of different plant parts is given in Table 2. The extractive yield varied among the different plants and also among the solvents used. Petroleum ether and ethyl acetate extract showed less extractive yield as compared to aqueous and methanol extracts. Among the plants screened maximum yield was in methanolic extract of *G. ulmifolia* (11.28%) and minimum extractive yield was in petroleum ether extract of *S. cumini* (0.36%).

**TABLE 2:** EXTRACTIVE YIELD (%) OF DIFFERENT PLANTS IN DIFFERENT SOLVENTS WITH INCREASING POLARITY

Botanical Name	Parts used	Extracts	% Yield (w/w)
*G. ulmifolia*	Leaf	Petroleum ether	0.64
		Ethyl acetate	0.77
		Methanol	7.27
		Aqueous	11.28
*M. zapota*	Leaf	Petroleum ether	4.21
		Ethyl acetate	1.45
		Methanol	10.86
		Aqueous	3.78
*M. azedarach*	Leaf	Petroleum ether	0.79
		Ethyl acetate	1.69
		Methanol	7.14
		Aqueous	10.65
*M. azedarach*	Stem	Petroleum ether	0.48
		Ethyl acetate	0.62
		Methanol	2.97
		Aqueous	2.87
*S. cumini*	Leaf	Petroleum ether	0.36
		Ethyl acetate	1.77
		Methanol	8.23
		Aqueous	2.96
*W. tomentosa*	Leaf	Petroleum ether	0.69
		Ethyl acetate	1.32
		Methanol	5.11
		Aqueous	7.33
*W. tomentosa*	Stem	Petroleum ether	1.33
		Ethyl acetate	0.81
		Methanol	3.10
		Aqueous	3.28
*W. tomentosa*	Fruit	Petroleum ether	3.32
		Ethyl acetate	2.81
		Methanol	7.86
		Aqueous	7.86

The crude powder of all the eight plant parts were subjected to preliminary phytochemical analysis and the results are shown in Table 3. All the eight plant parts showed the presence of triterpenes and cardiac glycosides in higher amount while other phytoconstituents were present in trace amount or absent. It is possible that these secondary metabolites might be responsible for the bioactivity of the plant extracts[28].

**TABLE 3:** PRELIMINARY PHYTOCHEMICAL ANALYSES OF MEDICINAL PLANTS SCREENED

Plant name	Parts used	Alk			Fla	Tan	Phl	Tri	Ste	Sap	Car
									
		Dra	May	Wag							
*G. ulmifolia*	Leaf	−	−	+	−	+	−	+	+	+	+++
*M. zapota*	Leaf	−	−	+	+	+++	++	+++	−	++	+
*M. azedarach*	Leaf	−	+	−	−	+	−	++	+++	+	+++
*M. azedarach*	Stem	−	++	+++	−	−	−	+	−	−	++
*S. cumini*	Leaf	−	+	−	−	+++	+	+++	+	++	−
*W. tomentosa*	Leaf	−	+	+	−	+	+++	+++	++	+	+++
*W. tomentosa*	Stem	−	++	+++	−	−	−	+	−	+	+++
*W. tomentosa*	Fruit	−	−	+++	+	−	−	+	+++	−	+++

− : No presence;

+ : Less presence;

++ : Moderate presence;

+++ : High presence; Alk: Alkaloids; Dra: Dragendroff; May: Mayer; Wag: Wagner; Fla: Flavonoids; Tan: Tannins; Phl: Phlobatannins; Tri: Triterpenes; Ste: Steroids; Sap: Saponins; Car: Cardiac glycosides

The antibacterial activity of eight parts of five plants belonging to five different families was assayed *in vitro* by agar well diffusion method against five different bacterial strains. The parts used for the study were leaf, stem and fruit, while the solvents used were petroleum ether, ethyl acetate, methanol and water. Therefore, in all, 32 extracts were evaluated for antibacterial activity as shown in Table 4. Aqueous extract did not show any antibacterial activity against almost all the bacteria studied. Similar results have been reported in the literature[29,30]. Among the plants screened, *W. tomentosa* leaf and fruit showed best antibacterial activity. Petroleum ether extracts of almost all plants showed antibacterial activity against both Gram-positive bacteria while they were inactive against Gram-negative bacteria. Highest antibacterial activity was shown by ethyl acetate extract of *W. tomentosa* leaf against *S. aureus* and by petroleum extract of *S. cumini* against *B. subtilis*. The most susceptible bacterium was *S. aureus*, while the most resistant bacteria were *S. typhimurium* and *E. aerogenes*.

**TABLE 4:** ANTIMICROBIAL ACTIVITIES OF MEDICINAL PLANTS SCREENED

Plant Name(Used Parts)	Extracts	Bacteria				
		
		*BS*	*SA*	*PA*	*ST*	*EA*
*G. ulmifolia (Leaf)*	PE	11±0.17	14±0.33	-	-	-
	EA	13±0.29	-	-	-	-
	ME	-	-	-	-	-
	AQ	-	-	14±0.33	-	-
*M. zapota (Leaf)*	PE	-	14±0.17	-	-	-
	EA	10±0.17	-	-	-	-
	ME	12±0.33	12±0.5	14±0.29	13±0.17	11±0.17
	AQ	-	-	-	-	-
*M. azedarach* (Leaf)	PE	12±0.33	-	-	-	-
	EA	-	12±0.33	-	-	-
	ME	12±0.33	12±0.73	-	-	-
	AQ	-	-	-	-	-
*M. azedarach* (Stem)	PE	11±0.58	15±0.33	-	-	-
	EA	11±0.17	14±0.73	-	-	-
	ME	-	-	-	-	-
	AQ	-	-	12±0.33	-	-
*S. cumini* (Leaf)	PE	19±0.58	-	-	-	-
	EA	15±0.17	-	-	-	-
	ME	12±0.33	13±0.88	12±0.50	12±0.17	-
	AQ	-	-	-	11±0.29	-
*W. tomentosa* (Leaf)	PE	11±0.33	13±0.58	-	-	-
	EA	12±0.33	20±1.45	-	-	-
	ME	-	16±0.58	-	-	-
	AQ	-	-	-	-	-
*W. tomentosa* (Stem)	PE	11±0.17	11±0.17	-	-	-
	EA	-	13±0.33	-	-	-
	ME	-	-	10±0.17	-	-
	AQ	-	-	11±0.33	-	-
*W. tomentosa* (Fruit)	PE	-	-	-	-	-
	EA	11±0.58	16±0.58	-	-	12±0.29
	ME	11±0.33	15±0.17	11±0.29	-	11±0.17
	AQ	-	-	-	-	-

*BS = Bacillus subtilis; SA = Staphylococcus aureus; PA = Pseudomonas aeruginosa; ST = Salmonella typhimurium; EA = Enterobacter aerogenes;* PE = Petroleum ether; EA = Ethyl acetate; ME = Methanol; AQ = Aqueous; - means no activity; Values are Mean ± SEM, n=3

From the above results, it can be concluded that Gram-positive bacteria are susceptible to plant extracts more as compared to Gram-negative bacteria. Various workers have already reported similar results[31–33]. The difference in sensitivity might be ascribed to the difference in morphological constitutions between Gram-positive and Gram-negative organisms. Many plant species present inhibition zones of differing diameters; however, size difference of the inhibition zone depends primarily upon many factors for e.g. diffusion capacity of substances (present in the extracts) in the agar medium, antimicrobial activity of diffused substances, growth and metabolic activity of microorganisms in the medium. Inhibition zone diameter can further be associated with polarities of substances which make up the tested extracts and also with cell wall composition of test organisms since Gram-positive bacteria present cell walls with lower lipid levels than do Gram-negative bacteria[34].

Plant phenolics constitute one of the major groups of compounds acting as primary antioxidant free radical terminators. These compounds possess a wide spectrum of chemical and biological activities including radical scavenging properties. Several studies have described the antioxidant properties of medicinal plants, foods and beverages which are rich in phenolic compounds[35–37].

The total phenol content of eight plant parts is shown in Table 5. Amongst the four extracts, methanol extracts showed higher amount of phenolic content followed by aqueous extracts, while petroleum ether extracts showed trace amount of phenolic content. Highest amount of total phenol content was in methanol extracts of *M.* zapota (194.06) and *S. cumini* (179.89). The flavonoid content of eight plant parts is shown in Table 6. Amongst the four extracts, petroleum ether extracts showed higher amount of flavonoid content, while aqueous extracts showed trace amount of flavonoid content.

**TABLE 5:** TOTAL PHENOL CONTENT (MG/G) OF MEDICINAL PLANTS SCREENED

Plant Name	Parts Used	Extracts			
		
		Petroleum ether	Ethyl acetate	Methanol	Aqueous
*G. ulmifolia*	Leaf	5.01±0.17	6.68±0.23	16.60±0.20	19.35±0.17
*M. zapota*	Leaf	1.67±0.31	18.76±0.28	194.06±1.21	88.92±0.34
*M. azedarach*	Leaf	0.39±0.03	9.92±0.12	50.01±0.71	38.22±0.56
*M. azedarach*	Stem	3.83±0.09	5.99±0.35	21.71±0.20	47.65±0.13
*S. cumini*	Leaf	3.40±0.20	7.46±0.28	179.89±0.64	93.54±0.42
*W. tomentosa*	Leaf	0.49±0.07	10.21±0.10	54.82±0.62	27.31±0.20
*W. tomentosa*	Stem	3.34±0.06	10.31±0.15	51.09±0.27	50.60±0.09
*W. tomentosa*	Fruit	1.37±0.26	6.9±0.30	36.25±0.27	38.22±0.17

Values are Mean ± SEM, n=3

**TABLE 6:** FLAVONOID CONTENT (MG/G) OF MEDICINAL PLANTS SCREENED

Plant Name	Parts Used	Extracts			
		
		Petroleum ether	Ethyl acetate	Methanol	Aqueous
*G. ulmifolia*	Leaf	15.54±0.44	24.07±1.50	16.14±1.17	1.624±0.22
*M. zapota*	Leaf	39.01±1.14	5.21±0.57	35.55±0.21	0.715±0.05
*M. azedarach*	Leaf	12.79±0.37	7.24±0.26	21.90±0.05	15.78±0.22
*M. azedarach*	Stem	38.08±0.89	19.65±0.12	7.59±0.09	7.41±0.02
*S. cumini*	Leaf	22.19±0.37	1.89±0.33	28.38±0.32	3.4±0.16
*W. tomentosa*	Leaf	28.93±0.14	4.91±0.20	8.21±0.22	17.41±0.08
*W. tomentosa*	Stem	54.40±0.03	53.87±0.24	5.54±0.15	5.58±0.03
*W. tomentosa*	Fruit	33.36±0.27	14.74±0.55	3.35±0.09	4.49±0.17

Values are Mean ± SEM, n=3

Various assays are used to test for antioxidant activity but the mostly widely used methods are those that involve generation of free radical species which are then neutralized by antioxidant compounds[38,39]. DPPH radical is commonly used as a substrate to evaluate antioxidant activity. In the present work, eight parts of five plant species, in various solvents were evaluated for their free radical scavenging activity. Out of 32 extracts investigated, 19 extracts showed IC_50_ value more than 1000 μg/ml, while the remaining 13 plant extracts showed a varied level of DPPH scavenging activity. IC_50_ values ranged from 24.5 μg/ml to 800 μg/ml (Table 7). Ascorbic acid was used as standard and its IC_50_ value was 11.4 μg/ml. The lowest IC_50_ value was of leaf of *M. zapota* in methanol extract (IC_50_ = 24.5 μg/ml) and the highest IC_50_ value was of leaf of *G. ulmifolia* (IC_50_ = 655 μg/ml). Amongst all the four extracts, methanol extract showed better antioxidant activity.

**TABLE 7:** DPPH FREE RADICAL SCAVENGING ACTIVITY IN VARIOUS SOLVENT EXTRACTS OF DIFFERENT PLANTS

Plant Name	Parts Used	IC_50_ Value in ug/ml			
		
		Petroleum ether	Ethyl acetate	Methanol	Aqueous
*G. ulmifolia*	Leaf	>1000	>1000	655	>1000
*M. zapota*	Leaf	>1000	320	24.5	87.5
*M. azedarach*	Leaf	>1000	>1000	610	400
*M. azedarach*	Stem	>1000	>1000	>1000	800
*S. cumini*	Leaf	>1000	>1000	49	85
*W. tomentosa*	Leaf	>1000	>1000	530	205
*W. tomentosa*	Stem	>1000	>1000	460	360
*W. tomentosa*	Fruit	>1000	>1000	>1000	>1000

Natural extracts with proven antioxidant activity are usually composed with their phenolic moiety, for example flavonoids, coumarins and tocopherols. Organic acids, carotenoids and tannins can also be present and act as antioxidants or have a synergistic effect with phenolic compounds[40]. However in present work, methanol extract of *M. zapota* showed promising result in total phenol content and antioxidant activity, while in other plant extracts there was weak correlation between phenolic content and antioxidant potency. This suggests that non-phenolic compounds may also be responsible for the observed antioxidant activity as also suggested by Yam *et al*[13].

In the present study, antimicrobial and antioxidant activity of eight plant parts were screened. Five bacterial strains were used for the antimicrobial activity. Amongst all the plants screened, *W. tomentosa* leaf and fruit showed best antibacterial activity; while *M. zapota* showed best DPPH free radical scavenging activity in methanol extract. The Gram-positive bacteria were more susceptible than Gram-negative bacteria. Amongst the four extracts used, methanol extracts followed by ethyl acetate extracts showed better antimicrobial activity. Highest total phenol content was shown by *M. zapota* and *S. cumini* in methanol extract, while highest flavonoid content was shown by *W. tomentosa* stem in petroleum ether extract and ethyl acetate extract. In all the plants, cardiac glycosides and triterpenes were more as compared to other phytoconstituents.

Based on these results, it can be concluded that plant extracts have great potential as antimicrobial compounds against microorganisms and they can be used in the treatment of infectious diseases caused by resistant microorganisms. They can also be a source of natural antioxidants. Due to their antibacterial and antioxidant activities *W. tomentosa* and *M. zapota* extracts have promising potential as a source of natural antioxidant and antimicrobial agents. Such screening of various natural organic compounds and identification of active agents is the need of the hour because successful prediction of lead molecule and drug-like properties at the onset of drug discovery will pay off later in drug development.
